# Unusual visual impairment after enhancement refractive surgery

**DOI:** 10.1093/jscr/rjae074

**Published:** 2024-02-16

**Authors:** Xia Li, Yong Gu

**Affiliations:** Department of Ophthalmology, Shanghai Aier Eye Hospital, Shanghai 200031, PR China; Shanghai Aier Eye Institute, Shanghai 200031, PR China; Department of Ophthalmology, Wu Xi Aier Eye Hospital, Wuxi, Jiangsu 214000, PR China

**Keywords:** enhancement surgery, flap lift, epithelial in-growth, visual impairment

## Abstract

We reported a case of rapidly developed corneal epithelial in-growth (EI) deteriorating visual acuity (VA) within the initial postoperative day. A 37-year-old male presented with decreased VA for 2 years. He underwent LASIK surgery 13 years ago. After enhancement surgery, postoperative VA was much worse than preoperative best corrected visual acuity (BCVA) 20/20 and decreased rapidly. VA of OD was 20/40 on Day 1, and 20/70 on Day 5, OS 20/20 on Day 1, 20/25 on Day 10, and 20/50 on Day 13 postoperatively. Corneal topography and optical coherence tomography (OCT) showed distinctive features. The patient was diagnosed with corneal EI postoperatively. After scraping ectopic corneal epithelial cells, the cornea became transparent and VA improved. Despite its rarity, early postoperative EI can occur within 1 day after enhancement surgery and can progress rapidly. OCT and corneal topography provide distinctive manifestations aiding diagnosis.

## Introduction

Corneal epithelial in-growth (EI) is extremely uncommon in the immediate postoperative period. To our knowledge, this is the first reported case of EI occurring on the first day after surgery causing a significant decrease in visual acuity (VA).

## Case report

A 37-year-old male presented with decreased VA for 2 years. He had undergone LASIK surgery 13 years ago. His uncorrected distance visual acuity (UCVA) was right eye (OD) 20/100, and 20/100 left eye (OS), improving to 20/20 with a refractive correction of OD -4.00DS, and OS -5.00DS/−0.75DCx10°. The patient underwent uneventful flap-lift enhancement surgery. After enhancement surgery, postoperative VA was much worse than preoperative best corrected visual acuity (BCVA) 20/20 and decreased rapidly. One day postoperatively, VA was 20/40 OD with a refractive correction −0.50DS/−0.50 DC → 0.7. VA was 20/20 OS. Intraocular pressure by non-contact tonometry was 11 mmHg OD, 11 mmHg OS.

**Figure 1:**
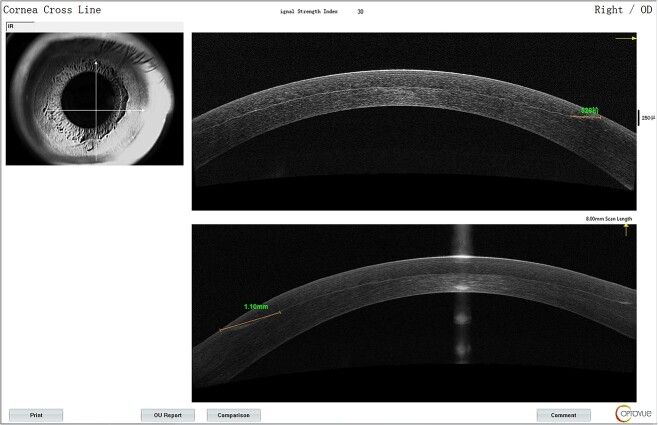
AS-OCT signs for the right eye on the first postoperative day.

The lower part of the flap appeared grayish-white from Day 1.On AS-OCT, the lesion extent was 1.1 mm from the edge (Fig. 1) with reflectivity becoming higher. It was deemed as corneal edema and postoperative immunological response. The patient was prescribed fluorometholone 0.1% eye drops and 0.3% Ofloxacin eye drops for both eyes and asked to return in 5 days for reevaluation.

**Figure 2:**
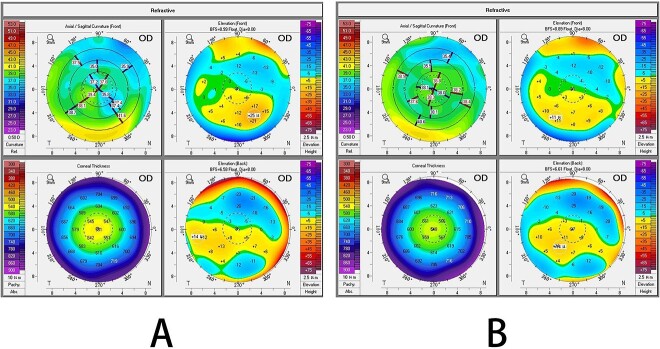
Corneal topography after enhancement surgery: (A) first postoperative day; (B) fifth postoperative day.

The VA in the right eye decreased to 20/70 on Day 5. On slit-lamp examination, interface lesions resembling water or oil stains were present at the lower flap edge. On corneal topography, the anterior elevation of the nasal inferior quadrant increased over time with thickening in the same area. The irregular oblique astigmatism was 0.6 D on Day 1, 2.0 D on Day 5 (Fig. 2).

**Figure 3:**
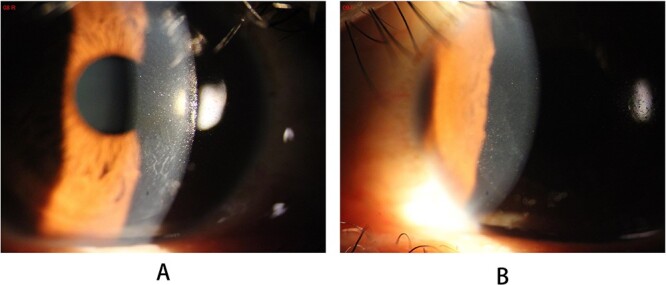
Slit-lamp photography of the lesions on the 13th postoperative day: (A) right eye; (B) left eye.

**Figure 4:**
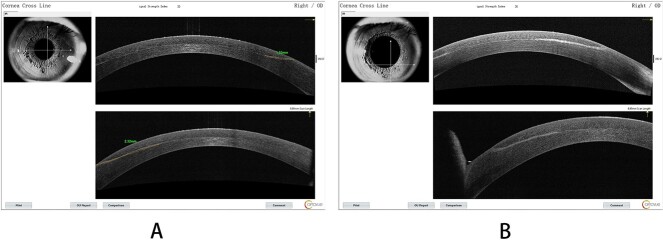
(A) AS-OCT signs for the right eye on the fifth day; (B) a detailed shot of the nasal inferior quadrant region.

The VA in the patient’s left eye exhibited a marked decrease, from an initial measurement of 20/25 on Day 12, to 20/50 just a day later. Concurrently, the VA in the right eye remained 20/70 on Day 13. Distinct interface lesions were observed in the nasal inferior quadrant of both eyes, spreading radially toward the pupil and originating at the lower edge of the corneal flap. This pattern was consistently observed over the 13-day monitoring period for both eyes (Fig. 3). On AS-OCT, the lesions were seen to enlarge in extent, elongating up to 2.32 mm toward the pupil by Day 13, exhibiting a higher level of reflectivity (Fig. 4).

Reflecting on the enhancement surgery process, the corneal flap was found to be relatively thick and somewhat challenging to fold. The flap thickness was 257 um OD and 265 um OS on AS-OCT. The epithelial cells displayed a looser connection with the membrane layer.

With distinctive features and onset progression, the patient was diagnosed with corneal EI and loose epithelial tissue was noted to be inserted into the interface during surgery for the right eye. A surgical scraping procedure was performed on Day 13 (Video S1, Supplementary Data). A thick epithelial sheet was found spreading from the flap edge. The patient was satisfied with clear vision of 20/25 OU on the first postoperative day. The cornea became transparent.

## Discussion

Corneal EI is extremely uncommon in the immediate postoperative period. Upon thorough literature review, no patients presented with severe EI on the first postoperative day. This added difficulties to diagnosis in the first few days. Christopher J [1] reported 46 patients (55 eyes) out of 305 undergoing LASIK developed EI, with onset intervals 0.5–108 months post-surgery. The EI was primarily peripheral in 49 eyes (89%) and central in 6 eyes (11%). Thirty-five eyes (64%) generally showed mild EI (<1 mm from the flap edge). The epithelial cells grew slowly over time.

The clinical manifestations where inserted epithelial followed the rapidly growing cells were totally different from the common EI. During surgery, the epithelial layer was loose and partly dissected from the basement membrane. The entire epithelial layer was inserted into the interface through the incision. The driving force for growth stemmed from limbal stem cells, with a growth rate approximating that of normal epithelial cells.

The thickness of the entire epithelial cell layer changed corneal shape and regularity, interfering with VA in the early postoperative stage and unable to be corrected with spectacles.

The inserted corneal epithelial layer interfered with corneal incision healing. From the flap lift scraping procedure (Video S1), the incision didn’t heal normally and was easily separated.

Anterior basement membrane dystrophy (ABMD) was a risk factor for the EI. Of patients with ABMD, 25.7% had some degree of EI in primary cases [2]. In normal patient population, EI incidence was 0% [3] to 2% [4] in primary cases and 32% in flap lift enhancements [5]. This loose epithelium could reattach or slough off. If sloughing occurred, it could lead to flap edema, causing elevation of the flap margin and opening up a potential space within the interface for epithelial cell migration from the flap perimeter. As a result, these patients faced an elevated risk of EI [6, 7].

A thin flap was previously identified as a risk factor for EI [8]. However, the flap in this case was unusually thick, exceeding 250 um in both eyes. Given the thickness of the flap, why did EI occur in this case? When the flap thickness was within normal range, EI was more likely to occur in eyes with thinner flaps (126.0 ± 29.1 um) compared with eyes with thicker flaps (133.8 ± 27.3 um) [4]. In cases with extremely thick flaps, like this case, the flap may be less elastic and more difficult to achieve optimal incision apposition. This irregularity could promote EI.

It was rare for the epithelial layer to insert into the interface during surgery and it took several days to make the EI diagnosis. Two factors likely contributed to epithelial cell insertion in this case: loose epithelial cells; and an irregular incision due to the thick flap. The inserted full-thickness epithelial layer grew rapidly, interfering the VA seriously. On Day 13, the definitive diagnosis was made and the scraping procedure was performed. The in-growth epithelial cells were completely removed as well as the epithelial cells 1–2 mm outside the incision. There was no EI recurrence and the VA kept normal and steady after the procedure.

## Supplementary Material

Video1_rjae074

## Data Availability

The datasets used and/or analysed during the current study are available from the corresponding author on reasonable request.
